# Transcriptome Analysis Revealed Plant Hormone Biosynthesis and Response Pathway Modification by *Epichloë* *gansuensis* in *Achnatherum* *inebrians* under Different Soil Moisture Availability

**DOI:** 10.3390/jof7080640

**Published:** 2021-08-06

**Authors:** Zhenrui Zhao, Mingzhu Kou, Rui Zhong, Chao Xia, Michael J. Christensen, Xingxu Zhang

**Affiliations:** 1 State Key Laboratory of Grassland Agro-Ecosystems, Key Laboratory of Grassland Livestock Industry Innovation, Ministry of Agriculture and Rural Affairs, College of Pastoral Agriculture Science and Technology, Lanzhou University, Lanzhou 730020, China; zhaozhr16@lzu.edu.cn (Z.Z.); koumzh18@lzu.edu.cn (M.K.); zhongr12@lzu.edu.cn (R.Z.); xiac@lzu.edu.cn (C.X.); 2 Grasslands Research Centre, Private Bag 11-008, Palmerston North 4442, New Zealand; mchristensenpn4410@gmail.com

**Keywords:** *Epichloë gansuensis*, *Achnatherum inebrians*, soil moisture availability, plant hormones, transcriptome analysis, pathway

## Abstract

The present study was designed to explore the effects of the endophyte *Epichloë gansuensis* on gene expression related to plant hormone biosynthesis and response pathways and the content of salicylic acid (SA) and jasmonic acid (JA) hormones of *Achnatherum inebrians*, under different moisture conditions. Through a pot experiment and transcriptome analysis, we found a total of 51 differentially expressed genes (DEGs) related to hormone biosynthesis and response pathways, including 12 auxin related genes, 8 cytokinin (CTK) related genes, 3 gibberellin (GA) related genes, 7 abscisic acid (ABA) related genes, 7 ethylene (ET) related genes, 12 JA related genes and 4 SA related genes. Furthermore, key genes of JA and SA biosynthesis and response pathways, such as *LOX2S*, *AOS*, *OPR*, *ACX*, *JMT*, *JAZ*, *PAL*, *NPR1*, *TGA* and *PR-1*, showed different degrees of upregulation or downregulation. Under 60% soil moisture content, the JA content of endophyte-free (EF) *A. inebrians* was significantly (*p* < 0.05) higher than that of endophyte-infected (EI) *A. inebrians*. Under 30% and 60% soil moisture content, the SA content of EF *A. inebrians* was significantly (*p* < 0.05) higher than that of EI *A. inebrians*. SA content of EI *A. inebrians* under 30% and 60% soil moisture content was significantly (*p* < 0.05) higher than that under 15% soil moisture content. With both EI and EF plants, the SA and JA levels, respectively, are very similar at 15% soil moisture content. This study has revealed that *E. gansuensis* differentially activated plant hormone synthesis and signal transduction pathways of *A. inebrians* plants under different soil moisture availability.

## 1. Introduction

Due to a sessile lifestyle combined with unstable environments, plants inevitably encounter various biotic and abiotic stresses, such as diseases, pests, drought, salinity, extreme temperatures and heavy metals. Among these factors, drought is one of the most dramatic stresses that affects the normal growth and development of plants and limits crop yields [1,2]. In the past decade, global losses in crop production due to drought totaled about USD 30 billion [3]. Although twentieth-century trends in drought regimes are ambiguous, across many regions more frequent and severe droughts are expected in the twenty-first century [4]. To enhance resistance under adverse environments, plants form beneficial symbioses with a variety of organisms, such as mycorrhizal fungi, nitrogen-fixing bacteria and endophytic fungi [5,6,7].

Endophytic fungi of the genus *Epichloë* have been found in many cool season grasses [8,9]. *E. gansuensis* [10] or *E. inebrians* [11] are present in nearly 100% of *Achnatherum inebrians* plants in the arid and semi-arid grasslands of northwest China as the presence of an *Epichloë* endophyte in grasses can provide protection against abiotic and biotic stresses [12]. *Achnatherum inebrians* is a widespread perennial bunchgrass in the Qinghai–Tibet Plateau, including Tibet, Qinghai, Xinjiang and Gansu [13]. Studies have shown that the presence of *E. gansuensis* can improve the tolerance of *A. inebrians* to diseases [14], pests [15], low temperature [16], heavy metals [17] and drought [18].

Changes in levels of plant hormones indicate that they have an essential role in the response of grasses to infection with *Epichloë* endophytes. In *Festuca sinensis*, the content of gibberellin A3 (GA3), cytokinin (CTK) and indole-3-acetic acid (IAA) increased, whereas that of abscisic acid (ABA) decreased when infected with an *Epichloë* endophyte [19]. *Epichloë* endophyte infection increased the IAA content of leaves of *Stipa purpurea* [20]. *Lolium multiflorum* plants symbiotic with the endophyte *E. occultans* had lower concentrations of SA than did their non-symbiotic counterparts [21]. Xia [22] found that the content of ABA and IAA was increased while the CTK content was decreased when *A. inebrians* was infected with *E. gansuensis*.

Plants exposed to drought stress respond through changes in the levels of plant hormones. Drought-stressed Kentucky bluegrass (*Poa pratensis* L.) had a higher content of ABA and lower content of trans-zeatin riboside (ZR) and IAA in leaves, but a similar level of leaf gibberellin A4 (GA4) when compared to leaves of the well-watered control [23]. The content of ABA and IAA tended to increase in *Triticum aestivum* L. when subjected to water deficiency [24]. When two *Medicago sativa* cultivars, “San Isidro” and “Zhong Mu”, were faced with the challenge of drought stress, only the leaf ABA hormone content in leaves was increased, while the JA, SA, IAA, GA4 and ZR hormone content was unchanged [25]. Soil moisture content affected the content of ABA and CTK in leaves of *A. inebrians* [22]. However, only a few detailed studies have examined the key mechanisms at the gene level. Endophyte infection moderately increased transcription of *L. perenne* (perennial ryegrass) genes with roles in hormone biosynthesis and perception as well as in stress and pathogen resistance while reducing the expression of genes involved in photosynthesis [26]. Transcriptome analysis of *E. festucae*–*L. perenne* associations revealed dramatic changes in hormone biosynthetic and responsive gene expression [27]. Expression of genes encoding proteins involved in biosynthesis and signaling by SA was downregulated in the association of *L. perenne* plants with *E. festucae* endophytes [28]. Under drought stress, transcriptome analysis suggested that genes related to JA, IAA, ethylene (ET), ABA, CTK and GA were differentially expressed in *P. pratensis* [29]. At 15% and 30% relative soil moisture content, the presence of the *Epichloë* endophyte significantly affected the content of endogenous hormones of *A. inebrians* plants, including ABA, IAA and CTK as described by Xia [22].

*Achnatherum inebrians* plants grow in the grasslands of northwest China and are exposed to hash environmental conditions. For most of the growing season, the soil is low in water content. As nearly all *A. inebrians* plants in these grasslands are host to an *Epichloë* endophyte and because the presence of these endophytes increases tolerance to abiotic stresses, it seems likely the hormone-related defense system of plants would not adversely affect these mutualistic fungal symbionts. Thus, it is very likely that in dry conditions the levels of JA and SA, hormones that provide protection against abiotic and biotic stress, would be at a baseline level, able to respond to adverse abiotic and biotic stresses to provide protection to *A. inebrians* plants.

Consequently, the following hypothesis was established: firstly, in response to different soil moisture availability changes would occur in the SA and JA hormone content and in the expression level of genes associated with biosynthesis and response pathways of plant hormones, particularly SA and JA, in *A. inebrians* plants host to *E. gansuensis.* Secondly, endophyte-infected plants growing under the lowest soil moisture availability conditions would have the lowest levels of SA and JA. To test this hypothesis, we established endophyte-infected (EI) and endophyte-free (EF) *A. inebrians* plants in the greenhouse, then set a challenge of three soil moisture availabilities, including drought stress. To avoid the complication of above- and belowground fungal pathogens, and also insects and nematodes, the plants were grown in vermiculite that had been heat sterilized. The concentration of SA and JA hormones, the expression levels of genes associated with SA and JA biosynthesis and response pathways and also the expression levels of genes related to other plant hormones were measured in plants in each of the six treatments.

## 2. Materials and Methods

### 2.1. Plant Material and Experimental Design

Seeds were collected from EI and EF *A. inebrians* plants grown in the experimental field of the College of Pastoral Agriculture Science and Technology, Yuzhong Campus of Lanzhou University (104°39′ E, 35°89′ N, Altitude 1653 m) in 2013. Seed samples from each plant were maintained at 4 °C and were used for further study. We used seeds originating from a single EI and EF plant to reduce variability within our plant material at the start of the study. A pot experiment was carried out from 15 May to 15 July 2016 in the greenhouse of the College of Pastoral Agriculture Science and Technology, Yuzhong Campus of Lanzhou University. On 15 May, healthy-looking and well-filled seeds were sown in 120 pots (60 pots for EI plants and 60 pots for EF plants), with 3 seeds per pot (diameter: 24 cm; height: 15 cm), filled with vermiculite (75 g) that had been sterilized in an oven at 150 °C for 3 h. These pots were assigned at random to a position within a constant-temperature greenhouse (temperature: 26 ± 2 °C, moisture: 42 ± 2%) and watered sufficiently to keep the surface of the vermiculite moist. Following the appearance of the second fully expanded leaf, Hoagland’s solution was used to quantitatively water the pots every 7 days.

On June 10, watering of the pots was ceased to reduce the water-holding capacity (WHC) of each pot to 15% relative saturation moisture content (RSMC), a ratio of actual soil moisture content relative to potential maximum soil moisture saturation. On 15 June, 96 pots, 48 with EI and 48 with EF, that contained equal-sized *A. inebrians* plants were selected for the one-month trial of drought stress. Then, three different WHCs were established, including strong drought (D, 15% of WHC), and normal moisture (N, 30% WHC) or abundant moisture (W, 60% WHC) conditions, and for each WHC of both EI and EF plants there were 16 pots. During the trial, every evening at 6 o’clock, each pot was weighed, and water was added to maintain soil moisture content at 15%, 30% or 60% WHC, respectively. On 15 July, leaves from the plants of each treatment were separately collected and were immediately frozen in liquid nitrogen at −80 °C. Then the samples were stored in a refrigerator at −20 °C for subsequent transcriptome analysis and JA and SA content determination.

### 2.2. JA and SA Content Determination

The samples were sent to Shanghai Fanke Industrial Co., Ltd. (Shanghai, China) for the determination of JA and SA content by enzyme-linked immunosorbent assay (ELISA). First, the diluted samples and standards were added to the sample plates and reacted at 37 °C for 30 min. The plates were washed five times, enzyme-labeled reagent was added to plates with an ELISA instrument (MS, 352, Finland), reacted at 37 °C for 30 min and washed 5 times, then color developing solution was added and maintained at 37 °C for 10 min. Finally, termination solution was added and through the use of a spectrophotometer (DR6000, Hach, Loveland, CO, USA), the absorbance (OD value) of each sample was measured at the wavelength of 450 nm, and the content of JA and SA was calculated based on the OD values.

### 2.3. Transcriptome Analysis

For mRNA sequencing, a total amount of 3 μg RNA was isolated from the six treatments. Three biological replicates were used for each treatment. The concentration and integrity of RNA was assessed using a Qubit^®^ RNA Assay Kit in Qubit^®^2.0 Fluorometer (Life Technologies, Carlsbad, CA, USA) and an RNA Nano 6000 Assay Kit of the Agilent Bioanalyzer 2100 system (Agilent Technologies, Santa Clara, CA, USA).

Transcriptome analysis was performed by the Biomarker Technologies Company (Beijing, China). Sequencing libraries were generated using NEBNext^®^ Ultra^TM^ RNA Library Prep Kit for Illumina^®^ (NEB, San Diego, CA, USA). The library fragments were purified with an AMPure XP system (Beckman Coulter, Beverly, CA, USA). Afterward, the libraries were sequenced on an Illumina HiSeq 2500 platform.

Gene function was annotated based on the following databases: NR (NCBI non-redundant protein sequences); Pfam (Protein family); KOG/COG/eggNOG (Clusters of Orthologous Groups of proteins); Swiss-Prot (a manually annotated and reviewed protein sequence database); KEGG (Kyoto Encyclopedia of Genes and Genomes) and GO (Gene Ontology). The expression level of each gene was calculated using FPKM (fragments per kilobase per million mapped fragments). Differential expression analysis was performed using DEseq2_EBseq with false discovery rate (FDR) ≤ 0.05 and FC ≥ 2. GO enrichment analysis of the differentially expressed genes (DEGs) was performed by the GOseq R package based on a Kolmogorov–Smirnov test [30], and the statistical enrichment of DEGs in the KEGG pathway was determined by KOBAS software [31].

All raw sequences used in this study have been deposited in Sequence Read Achieve (SRA) of the NCBI database under the accession number PRJNA748183.

### 2.4. Quantitative Real-Time PCR (qRT-PCR) Analysis

To synthesize cDNA, total RNA from diluted stocks of the same RNA that was subjected to RNA-seq was used in each reverse transcription reaction using the RevertAid First Strand cDNA Synthesis Kit (Thermo Scientific). qRT-PCR reactions were analyzed in StepOnePlus^TM^ Real-Time System (Applied Biosystems, Waltham, MA, USA) with SYBR^®^ Select Master Mix (2X) (Applied Biosystems, Waltham, MA, USA). The primers listed in Supplementary Table S1 were synthesized by Wuhan Tianyi Huiyuan Biotechnology Co., Ltd. The qRT-PCR analysis of each sample was performed in triplicate. *Ubiquitin C* was used as internal reference, and the relative gene expression levels were calculated according to the 2^−ΔΔC^^T^ method [32].

### 2.5. Statistical Analyses

Differences of JA and SA content under different endophyte status and soil moisture levels were tested using two-way analysis of variance (two-way ANOVA) by SPSS 22.0 (SPSS Inc., Chicago, IL, USA). Significant differences of JA and SA content between EI and EF *A. inebrians* plants with the corresponding water treatment were examined by an independent-sample *t*-test. Fisher’s least significant differences (LSD) test was used to determine whether differences between means were statistically significant. In all tests, *p*-value < 0.05 was considered statistically significant.

## 3. Results

### 3.1. Differentially Expressed Gene (DEG) Analysis

**Figure 1:**
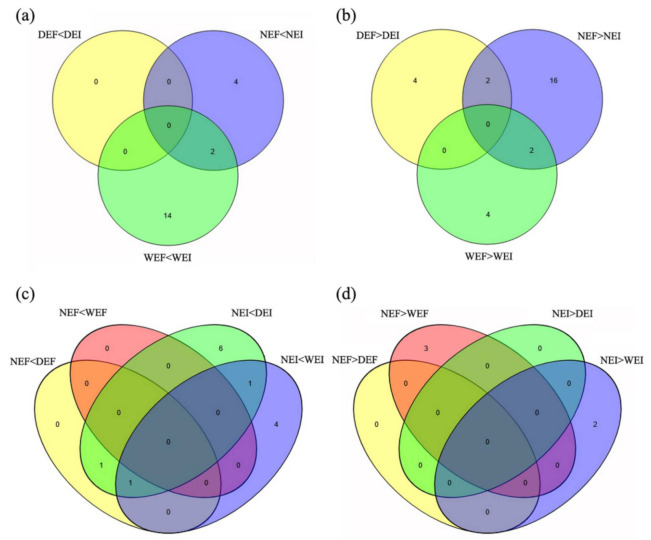
Numbers of upregulated and downregulated DEGs associated with the plant hormones under the status of *Epichloë gansuensis* (**a**,**b**) and different moisture content (**c**,**d**) in *Achnatherum inebrians* plants (D: drought, N: normal, W: well-watered, EI: endophyte infected and EF: endophyte free).

Upon comparison with the 30% soil moisture content treatment and the EF treatment at each soil moisture content, the unigenes with gene expression fold changes greater than or equal to 2 and with an FDR value below 0.05 were defined as DEGs. Based on these strict criteria, there were 6 (0 upregulated and 6 downregulated) DEGs between DEF versus DEI, 26 (6 upregulated and 20 downregulated) DEGs between NEF versus NEI, 22 (16 upregulated and 6 downregulated) DEGs between WEF versus WEI, 2 (2 upregulated and 0 downregulated) DEGs between NEF versus DEF, 3 (0 upregulated and 3 downregulated) DEGs between NEF versus WEF, 9 (9 upregulated and 0 downregulated) DEGs between NEI versus DEI and 8 (6 upregulated and 2 downregulated) DEGs between NEI versus WEI. At each treatment, we detected both unique and overlapping sets of DEGs (Figure 1).

### 3.2. DEGs Related to Hormone Biosynthesis and Response

**Figure 2:**
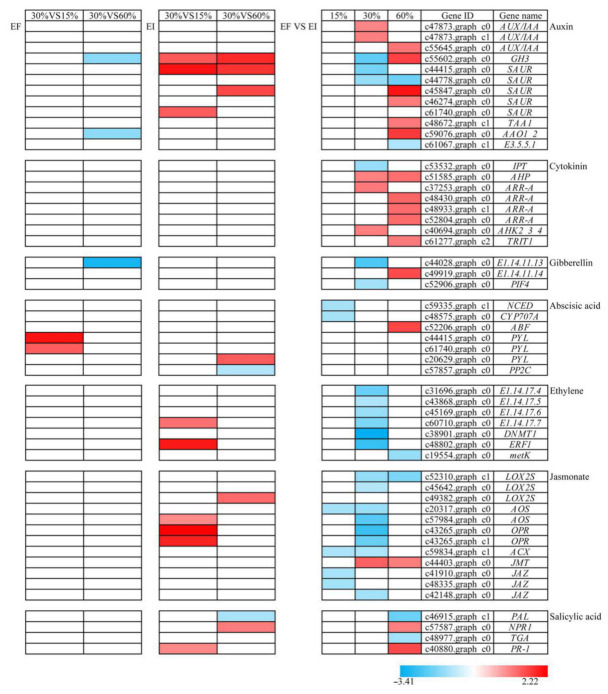
Transcript expression pattern of hormone biosynthesis and catabolism related genes in *Achnatherum inebrians* under different moisture content and the status of foliar *Epichloë gansuensis* endophyte (EI: endophyte infected and EF: endophyte free). Color bars ranging from blue to red represent downregulation and upregulation in transcript expression. Gene ID and gene name are shown.

Based on analysis under different treatments, we found a total of 51 DEGs related to hormone biosynthesis and response pathways (Figure 2), including 12 IAA related genes, 8 CTK related genes, 3 GA related genes, 7 ABA related genes, 7 ET related genes, 12 JA related genes and 4 SA related genes.

### 3.3. KEGG Pathway Enrichment Analysis of the DEGs

**Figure 3:**
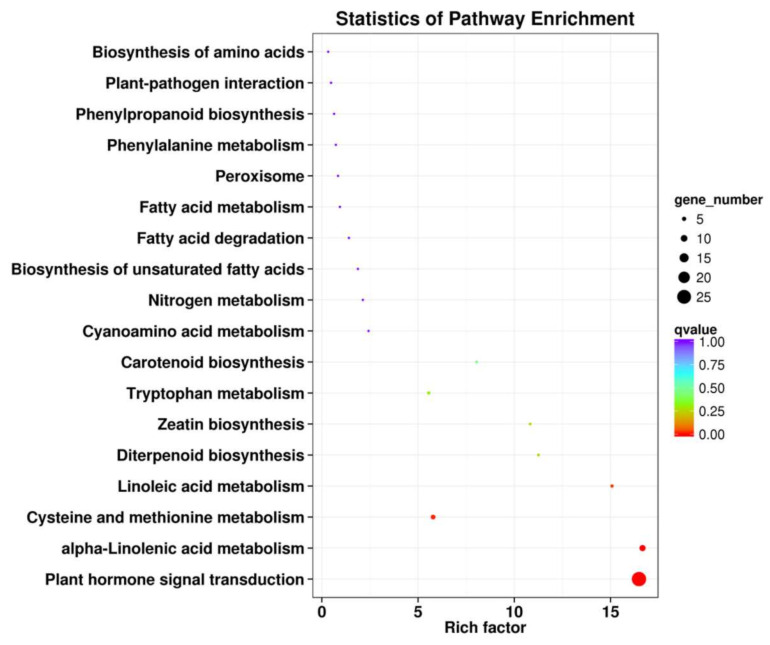
KEGG pathway enrichment of DEGs associated with *Achnatherum inebrians* under different moisture content and the status of *Epichloë gansuensis* endophyte. Only the top 20 most strongly represented pathways are displayed in the diagram. The q-value ranges from 0 to 1, and a q-value closer to 0 indicates greater enrichment.

To characterize the complex biological behavior of the transcriptome, 51 DEGs were subjected to a KEGG pathway enrichment analysis. The top 20 KEGG pathways with the highest representation of the DEGs are shown in Figure 3 The “Plant hormone signal transduction (ko04075)”, “alpha-Linolenic acid metabolism (ko00592)”, “Cysteine and methionine metabolism (ko00270)” and “Linoleic acid metabolism (ko00591)” categories were significantly enriched.

### 3.4. GO Functional-Enrichment Analysis of the DEGs

**Figure 4:**
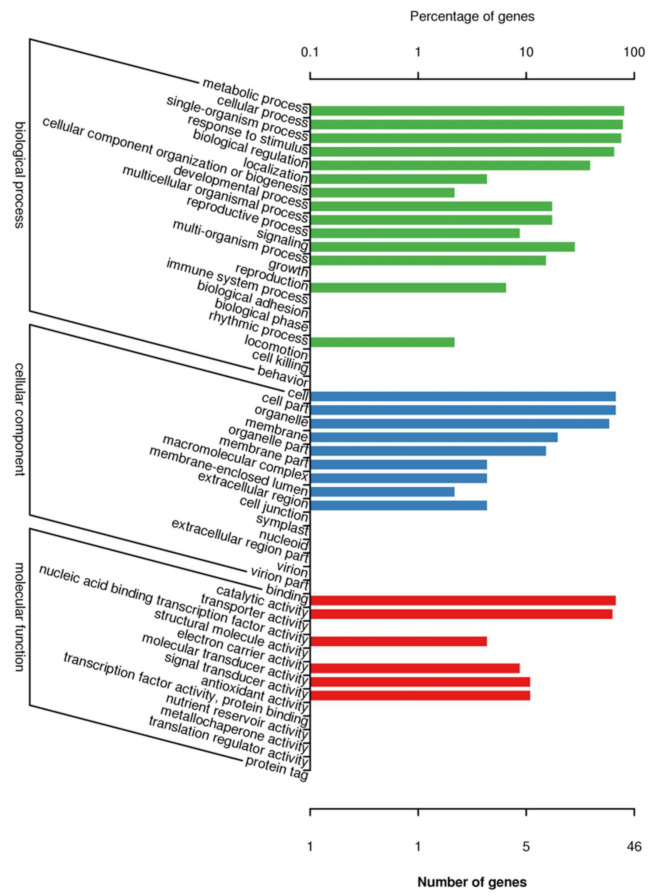
Go class of DEGs associated with *Achnatherum inebrians* under different moisture content and the status of *Epichloë gansuensis* endophyte.

A total of 29 GO categories were assigned to the 51 DEGs (Figure 4). GO term enrichment analysis categorized the annotated sequences into three main categories: biological process, cellular component and molecular function. In the biological process category, “metabolic process” was the most dominant group, followed by “cellular process” and “single-organism process”. Regarding the cellular component category, “cell” and “cell part” were the dominant categories, followed by “organelle”. In the molecular function category, “binding” was the most dominant group, followed by “catalytic activity”.

### 3.5. JA and SA Content

The presence or absence of the *Epichloë* endophyte and the interaction between soil moisture content and endophyte-infection had one significant effect on JA content (F_E_ = 22.585, *P*_E_ = 0.000; F_E*W_ = 4.904, *P*_E*W_ = 0.023). The JA content of EF *A. inebrians* was significantly (*p* < 0.05) higher than that of EI *A. inebrians* under 60% soil moisture content. Within EI or EF plants, soil moisture content had no significant (*p >* 0.05) effects on JA content. With EI plants, the levels of JA were similarly low at the three soil moisture content treatments. With the EF plants, only at 15% soil moisture content was the level of JA as low as for the EI plants.

**Figure 5:**
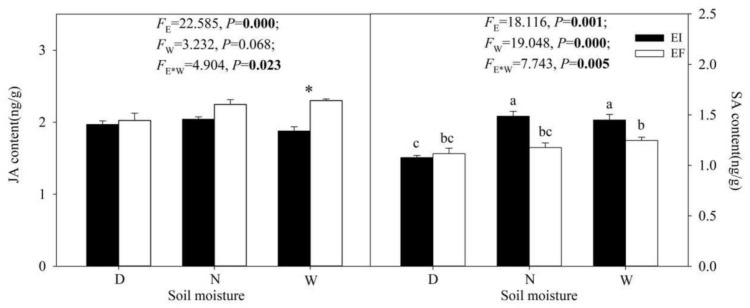
The JA and SA content under different moisture content and endophyte treatments (D: drought, N: normal, W: well-watered, EI: endophyte infected and EF: endophyte free). Values are means, with standard error bars (n = 3). The asterisk (*) means significant difference at *p* < 0.05 between EI and EF plants at corresponding water content. Columns with non-matching letters indicate a significant difference at *p* < 0.05.

Soil moisture content, the presence or absence of the *Epichloë* endophyte and the interaction between the two factors had significant effects on SA content (F_E_ = 18.116, *P*_E_ = 0.001; F_W_ = 19.048, *P*_W_ = 0.000; F_E*W_ = 7.743, *P*_E*W_ = 0.005). SA content of EF *A. inebrians* was significantly (*p* < 0.05) higher than that of EI *A. inebrians* under 30% and 60% soil moisture content. SA content of EI *A. inebrians* under 30% and 60% soil moisture content was significantly (*p* < 0.05) higher than that under 15% soil moisture content (Figure 5).

### 3.6. DEGs Related to Biosynthesis and Response Pathways of JA and SA

**Figure 6:**
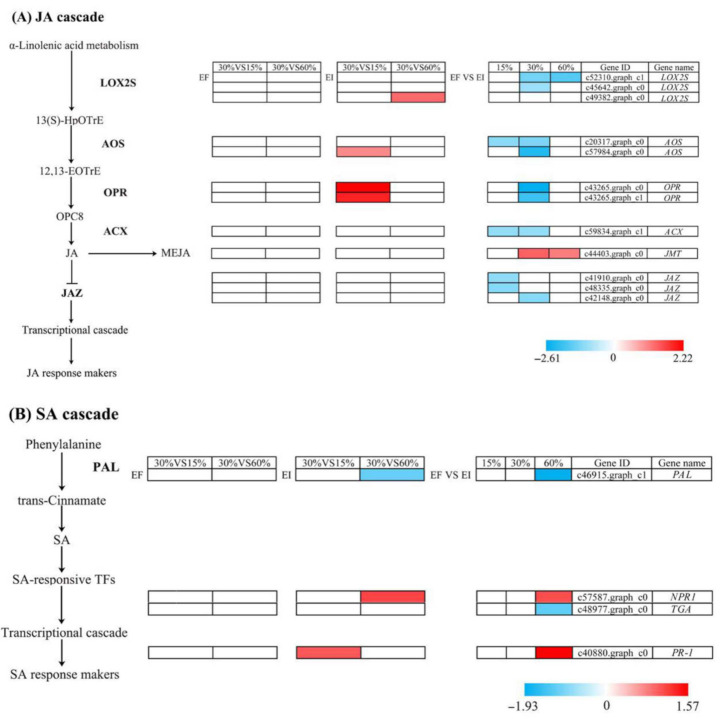
The cascade displaying synthetic and metabolic pathways of jasmonate (**A**) and salicylic acid (**B**) in *Achnatherum inebrians* under different moisture content and the status of foliar *Epichloë gansuensis* endophyte (EI: endophyte infected and EF: endophyte free). Color bars ranging from blue to red represent downregulation and upregulation in transcript expression. Gene ID and gene names are shown.

Based on analysis under different treatments, twelve genes involved in α-linolenic acid metabolism and JA-mediated signaling pathway and four genes involved in phenylalanine and SA-mediated signaling pathway were found. *AOS* (c57984.graph_c0) and *OPR* (c43265.graph_c0, c43265.graph_c1) expression levels were significantly (*p* < 0.05) upregulated in EI *A. inebrians* under 15% soil moisture content compared with those under 30% soil moisture content. *LOX2S* (c49382.graph_c0) expression level was significantly (*p* < 0.05) upregulated in endophyte-infected (EI) *A. inebrians* under 60% soil moisture content compared with that under 30% soil moisture content. *AOS* (c20317.graph_c0), *ACX* (c59834.graph_c1) and *JAZ* (c41910.graph_c0, c48335.graph_c0) expression levels were significantly (*p* < 0.05) downregulated under 15% soil moisture content in EI *A. inebrians* compared with those in EF *A. inebrians. LOX2S* (c52310.graph_c1, c45642.graph_c0), *AOS* (c20317.graph_c0, c57984.graph_c0), *OPR* (c43265.graph_c0, c43265.graph_c1), *ACX* (c59834.graph_c1) and *JAZ* (c42148.graph_c0) expression levels were significantly (*p* < 0.05) downregulated under 30% soil moisture content in EI *A. inebrians* compared with those in EF *A. inebrians. JMT* (c44403.graph_c0) expression level was significantly (*p* < 0.05) upregulated under 30% and 60% soil moisture content in EI *A. inebrians* compared with that in EF *A. inebrians. LOX2S* (c52310.graph_c1) expression level was significantly (*p* < 0.05) downregulated under 60% soil moisture content in EI *A. inebrians* compared with that in EF *A. inebrians* (Figure 6A).

*PR-1* (c40880.graph_c0) expression level was significantly (*p* < 0.05) upregulated in EI *A. inebrians* under 15% soil moisture content compared with that under 30% soil moisture content. *PAL* (c46915.graph_c1) expression level was significantly (*p* < 0.05) downregulated in endophyte-infected (EI) *A. inebrians* under 60% soil moisture content compared with that under 30% soil moisture content. *NPR1* (c57587.graph_c0) expression level was significantly (*p* < 0.05) upregulated in endophyte-infected (EI) *A. inebrians* under 60% soil moisture content compared with that under 30% soil moisture content. *PAL* (c46915.graph_c1) and *TGA* (c48977.graph_c0) expression levels were significantly (*p* < 0.05) downregulated under 60% soil moisture content in EI *A. inebrians* compared with those in EF *A. inebrians. NPR1* (c57587.graph_c0) and *PR-1* (c40880.graph_c0) expression levels were significantly (*p* < 0.05) upregulated under 60% soil moisture content in EI *A. inebrians* compared with those in EF *A. inebrians* (Figure 6B).

### 3.7. Validation of RNA-Seq Data Using qRT-PCR

**Figure 7:**
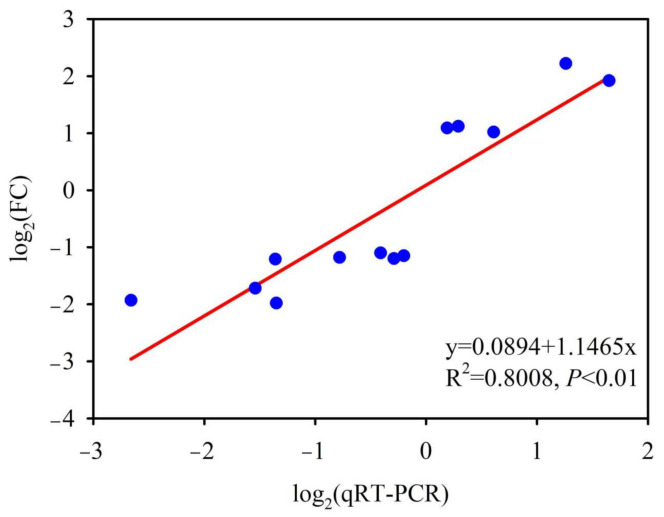
Validation of transcript expression changes by qRT-qPCR. The figure is based on log_2_ fold change of qRT-PCR and log_2_ fold change of RNA-seq. The linear trend line and the R^2^-value are shown.

The accuracy of RNA-seq results was confirmed using quantitative real-time polymerase chain reaction (qRT-PCR). Thirteen DEGs were selected for qRT-PCR analysis. Linear regression analysis detected a positive correlation between the qRT-PCR and RNA-seq results. In addition, the correlation coefficient was 0.8. Accordingly, the RNA-seq results were accurate and reliable in this study (Figure 7).

## 4. Discussion

We had predicted that, in response to different soil moisture availability, changes would occur in the SA and JA hormone content and in the expression level of genes associated with biosynthesis and response pathways of plant hormones, particularly SA and JA, in *A. inebrians* plants host to *E. gansuensis.* Our second major prediction regarding JA and SA was that endophyte-infected plants growing under the lowest soil moisture availability conditions would have the lowest levels of SA and JA. Our results support these predictions. The content of JA and SA did change with different soil moisture availability and the way these changes occurred differed with the presence or absence of *E. gansuensis. Achnatherum inebrians* plants in symbiosis with *Epichloë* endophytes had lower concentrations of JA than EF plants, and the difference was significant at 60% soil moisture content. Surprisingly, the lowest JA content of EI plants was with the highest soil moisture availability, which was contrary to our expectations that JA concentrations would increase as the soil moisture increased. In comparison, the levels of JA in EF plants increased as the soil moisture increased, and this is as we predicted. In contrast to JA, it was the levels of SA in EI plants that increased the most as the soil moisture content increased. With both EI and EF plants, the SA and JA levels, respectively, were very similar at 15% soil moisture content. In line with our prediction, the interaction between soil moisture content and *E. gansuensis* endophyte infection did alter the expressions of many plant-hormone-related genes, particularly JA- and SA-related genes.

In order to better understand the findings of this study, it is important to consider the nature and evolution of the relationship between the *Epichloë* endophyte and *A. inebrians*. *Epichloë gansuensis* is unique to *A. inebrians,* and their relationship would have co-evolved over a long period of time. In contrast to the host plant, the *Epichloë* endophyte is asexual and spreads clonally by colonizing the developing seed of the host grass. There will be continual selection for compatibility whenever endophyte-infected seeds germinate in the prevailing ecosystem. Any plant/endophyte association that does not grow well and/or add a competitive advantage will not persist. As nearly all *A. inebrians* plants growing in the arid, semi-arid grasslands in northwest China are host to an *Epichloë* endophyte, it is clear that the presence of the endophyte does provide a competitive advantage and is unlikely to be activating the host plant’s defense systems. That grasses can adversely react to the presence of an *Epichloë* endophyte has been shown in studies to establish novel endophyte grass associations by inserting mycelium from cultures of an *Epichloë* endophyte from one grass species into a slit adjacent to the shoot apex of seedlings growing in vitro of a different host species. This can result in a range of grass/*Epichloë* associations ranging from incompatible—host tissue or hyphal death, plant stunting and instability leading to the formation of endophyte-free tillers—to fully compatible [33,34,35]. In contrast to natural associations such as those of field-growing *A. inebrians*, in these incompatible novel associations, the plant defense system appears to have been activated. If the defense system has been activated in EI *A. inebrians* associations in these grasslands, it would appear that this does not adversely affect the endophyte.

Plant hormones play critical roles in responses to various adverse biotic and abiotic stresses. The drought-stress response in plants mainly involves the cross-talk between auxin, CTK, GA, ABA, ET, JA and SA.

Auxin, which is thought to regulate plant growth and development in diverse ways [36], participates in drought-stress responses at both physiological and molecular levels, including increases in auxin content and related gene expression [24,29,37,38]. Auxin function is manifested, at least in part, at the transcriptional level by regulating a group of primary responsive genes, including *Aux/IAAs*, *GH3s* and small auxin-up RNAs (*SAURs*) [39], which were up- or down regulated in our results. CTK synthesized by active root tips plays critical roles in the promotion of cell division, chloroplast differentiation and shoot development, counteraction of senescence and induction of photosynthesis gene expressions [40]. Drought stress inhibits CTK synthesis and accelerates CTK degradation, reducing CTK levels and gene expressions [22,23,29]. Moreover, CTK is found to increase cell membrane integrity and ABA hypersensitivity [41]. GAs promote various developmental processes throughout the plant life cycle, from seed germination, to leaf expansion, stem elongation, flower induction and development, to fruit setting and seed development [42]. Because the total effect of GAs on plant growth and elongation is opposite to that of water stress, GA levels and gene expressions levels often decrease under drought stress [29,43,44]. Typically, ABA is responsible for plant defense against drought stress, and water deficit is known to trigger an increase in ABA levels and gene expressions levels. Specifically, the accumulation of ABA promotes stomatal closure to minimize water loss, accelerates leaf senescence, downregulates plant growth and induces the biosynthesis of protective substances [41]. ET is produced in all plant tissues and plays an important role in many regulation pathways from seed germination to senescence and is also considered as a “stress hormone”, modifying responses to adverse conditions including drought stress [45]. *ERF* transcription factors have previously been shown to be important in mediating drought tolerance in plants [46]. In our study, expression levels of one *ERF1* gene (c48802.graph_c0) changed. JA and SA, generally considered to have antagonistic effects on each other, are signal substances closely related to the induction of insect resistance and disease resistance, respectively [21,47,48]. However, the role of JA and SA in drought resistance is unknown. Thus, the JA and SA pathways were considered to be a key focus of the present study.

Previous studies suggest that JA improves the drought resistance of plants by acting upstream of ABA [49,50]. Exogenous SA and methyl jasmonate (MeJA) application can enhance drought tolerance in chamomile plants [51,52]. In the JA biosynthesis pathway, lipoxygenases (LOXs), allene oxide synthase (AOS), 12-oxophytodienoate reductase 3 (OPR) and activity of an acyl-CoA oxidase (ACX) are key transcription factors activated under drought stress [53]. JA response is controlled by a group of nuclear proteins called jasmonate ZIM domain (JAZ) repressors. The expression of these genes was changed in this study. Most salicylic-acid-inducible genes are controlled by the transcriptional activator *NPR1,* and in our study, expression levels of one *NPR1* gene (c57587.graph_c0) was upregulated.

Endophytic fungi, beneficial microorganisms to plants, can generate changes in the plant immune responses [54]. Under drought stress, endophytic fungi can change the content of soluble sugar [55] and proline content [56] in host plants to maintain normal osmotic nodules in plants. Endophytic fungi can also increase antioxidant enzyme activity of host plants under drought stress [57] and produce antioxidants that scavenge reactive oxygen species [58] and enhance the antioxidant capacity of the host. The establishment of symbiotic relationships between plants and endophytic fungi entails changes in plant hormones when faced with drought stress. For example, a *Penicillium* endophyte and *Piriformospora indica* could promote the secretion of indole acetic acid (IAA) and gibberellin (GA) in the host plants to alleviate the harm of drought on the growth of host plants [59,60]. A previous study showed that the *Epichloë* endophyte of tall fescue (*F*. *arundinacea*) could also enhance production of IAA to promote the growth and development of host plants under drought stress [61]. An intriguing finding was that of Wang et al. [62], who found that pre-drought treatment led to significantly higher SA and lignin accumulation in EI *Leymus chinensis* in comparison with EF when exposed to the fungal pathogens *Curvularia lunata* and *Bipolaris sorokiniana*. In the recent study, Xia’s result [22] indicated that the endophyte significantly affected the content of endogenous hormones of *A. inebrians* plants, including ABA, IAA and CtK at 15% and 30% relative soil moisture content. Our study further demonstrated that in the symbiosis of *E. gansuensis* and *A. inebrians*, the content of JA and SA, as well as the activation of plant hormone synthesis is influenced by both the presence of the endophyte and the soil moisture content. However, the impact of the activation of these pathways, and specifically the role of JA and SA, on drought tolerance and the broader host defense responses, is still not fully established. To have a better understanding of how the presence of an *Epichloë* endophyte positively affects the persistence of *A. inebrians* plants growing in the arid, semi-arid grassland ecosystems of northwest China, further studies are required including detailed examination of the roots and leaves of field-growing EI and EF plants.

## Data Availability

All data supporting the findings of this study are available within the paper and within its Supplementary Materials published online. The RNA-seq used in this study have been deposited in the Sequence Read Achieve (SRA) of the NCBI database under the accession number PRJNA748183.

## Supplementary Materials

The following are available online at https://www.mdpi.com/article/10.3390/jof7080640/s1, Table S1: Differentially regulated unigenes associated with alpha-linolenic acid metabolism, plant hormone signal transduction and phenylalanine of endophyte-infected (EI) and endophyte-free (EF) *A. inebrians* under different moisture content identified in the RNA-seq analysis.
