# Supplementary material for: Transcriptome Analysis Revealed Plant Hormone Biosynthesis and Response Pathway Modification by Epichloë gansuensis in Achnatherum inebrians under Different Soil Moisture Availability
Source: J Fungi (Basel). 2021 Aug 6;7(8):640. doi: 10.3390/jof7080640 (PMC8398561; doi:10.3390/jof7080640)
Supplement: Supplementary file 1 [file jof-07-00640-s001.zip › jof-1255793-supplementary.pdf]

Supplementary Table S1 Differentially regulated unigenes associated with alpha-Linolenic acid metabolism - plant hormone signal transduction and phenylalanine of endophyte-infected (EI) and endophyte-free (EF) *A. inebrians* under different moisture content identified in the RNA-seq analysis.

Pathway	Name	Gene ID		Primers (5'-3')
alpha-Linolenic acid metabolism	*LOX2S*	c52310.graph_c1	Forward	CACGCCCAGCGTGTTTATGC
			Reverse	GGAGATGCTTGCAGGGCTCA
	LOX2S	c45642.graph_c0	Forward	AAGCAGCGAATGGAAAGCG
			Reverse	CGGAGACCGACCCAAGTAA
	LOX2S	c49382.graph_c0	Forward	GTCTTCGCTACCGTGCAAGC
			Reverse	AAGGGCCGAATCGCCATTGA
	AOS	c20317.graph_c0	Forward	GCGTCTGCTCCTACCTCGAC
			Reverse	AGTAGGCGTCGCCGATGAAC
	AOS	c57984.graph_c0	Forward	GGTGGTGGCTGACGAGTAGATT
			Reverse	GGCAGACACGGTTAAGGAGGA
	OPR	c43265.graph_c0	Forward	GTTCCTGGCGGCTTTCCTGA
			Reverse	CAAGGGCGCCGTCATCTTCT
	OPR	c43265.graph_c1	Forward	CCCTTGAACGCCTCCCTGTA
			Reverse	AACGACCACGGCATCCTCTA
	ACX	c59834.graph_c1	Forward	GCCAAGGACACGGAGTAGGC
			Reverse	CGTCGTCTCTGCGACCACAA
Plant hormone signal transduction	*JAZ*	c41910.graph_c0	Forward	CCACATCGCCATTCCCTCG
			Reverse	TGCTCAGCCGCTTCGTCA
	JAZ	c48335.graph_c0	Forward	CCCGTCCACCAAGGTCAAGG
			Reverse	GACGCCGTAACGGAGGTGTT
	JAZ	c42148.graph_c0	Forward	TTCTGCTCCGCCTTCACG
			Reverse	CCACCGCACCACAAACATC
	JMT	c44403.graph_c0	Forward	CTTCTCGCTGTTCCTTGC

			Reverse	GTCCCACATGCTGCTCAC
Phenylalanine	*PAL*	c46915.graph_c1	Forward	GCGCGAGATCAACTCCGTCA
			Reverse	GCGATCTCGGTGCCCTTGAA
Plant hormone signal transduction	*NPR1*	c57587.graph_c0	Forward	ACGCTTCTTCCCACGCTGTT
			Reverse	CCGGATGGCTCCGATTGAGG
	TGA	c48977.graph_c0	Forward	ATGGAGGGCATCGTCAGG
			Reverse	ACGGAGGCGGTGGAAGTAG
	PR-1	c40880.graph_c0	Forward	CATGCCGCCGGAGGGAATAA
			Reverse	TGGATGAACGTGGTGGCTTCT
